# Paraventricular Vitamin D Receptors Are Required for Glucose Tolerance in Males but Not Females

**DOI:** 10.3389/fendo.2022.869678

**Published:** 2022-05-10

**Authors:** Jessie Beck, Silvania da Silva Teixeira, Keisha Harrison, Gabrielle Phillips, Yanlin He, Stephanie Sisley

**Affiliations:** ^1^ Department of Pediatrics, Baylor College of Medicine, Houston, TX, United States; ^2^ Children’s Nutrition Research Center, Houston, TX, United States; ^3^ Pennington Biomedical Research Center, Brain Glycemic and Metabolism Control Department, Louisiana State University, Baton Rouge, LA, United States

**Keywords:** vitamin D receptor, glucose, brain, paraventricular hypothalamus, obesity

## Abstract

When delivered directly into the brain, vitamin D, can improve glucose levels in male mice. Additionally, the loss of the vitamin D receptor (VDR) in male mice’s paraventricular hypothalamus (PVH) results in impaired glucose tolerance. Data in humans shows that low vitamin D levels are detrimental to glucose homeostasis, an effect that may be more prominent in men. However, it is unknown if vitamin D action in the brain is required for normal glucose regulation in female mice. This study shows that in both viral and genetic models, male mice with obesity and PVH VDR loss have impaired glucose tolerance while female mice are unaffected. Weights were unaltered in both sexes by PVH VDR loss. Additionally, PVH VDR loss did not cause any glucose abnormalities in either sex when the mice were on a chow diet. Utilizing electrophysiology studies, we show PVH VDR loss resulted in decreased baseline firing frequency and resting membrane potential in males, but not females. Additionally, male mice with PVH VDR loss had impaired miniature excitatory postsynaptic currents (mEPSC), while females were unaffected. Interestingly, the PVH neurons of both sexes were activated by exogenous vitamin D (1,25-dihydroxyvitamin D3), an effect dependent upon the VDR. Thus, there is sexual dimorphism, for the actions of the PVH VDR on glucose regulation. PVH VDRs are necessary for normal glucose homeostasis in males but not females and this may be secondary to actions of the VDR on neuronal activity.

## Introduction

The association between vitamin D deficiency and diabetes has led several groups to test vitamin D as an anti-diabetic therapy in type 2 diabetes. However, the results have been confusing. Several studies have shown beneficial effects of vitamin D on measures of glucose control or insulin sensitivity (1–4) while others have found no effect (5–7). The factors mediating this profound heterogeneity are unknown.

Preclinical studies previously demonstrated that vitamin D action at the level of the pancreatic beta-cell was a clear pathway by which vitamin D could affect glucose homeostasis. Vitamin D receptors (VDRs) are present in pancreatic beta cells (8), are essential for beta cell survival (9), and the VDR-null mouse has impaired glucose tolerance with decreased insulin levels (10). Thus, the inconsistency of vitamin D supplementation to improve abnormal glucose tolerance is perplexing (1, 11–16).

Although previous work has focused on the effects of vitamin D in the periphery, the brain, specifically the hypothalamus, is well known to control peripheral blood glucose levels (17). We previously reported that VDRs have important actions in the brain to control glucose (18). Exogenous vitamin D delivered both into the third-ventricle of the hypothalamus and directly into the paraventricular hypothalamus (PVH) of the brain improves glucose tolerance in diet-induced obese (DIO) animals. More importantly, bilateral viral knockdown of the PVH VDR results in impaired glucose tolerance in DIO males.

Interestingly, recent human epidemiological studies have highlighted a sex-specific association of low vitamin D levels with glucose abnormalities. Furthermore, several epidemiological studies have shown that vitamin D deficiency is associated with insulin resistance only in males (19–22) or in postmenopausal females (23). However, whether there is a differential effect of VDR action in the brain in males vs. females has not been tested. Here, we aim to test the necessity of vitamin D receptors in the brain in both sexes in both *in vivo* and *ex vivo* experiments.

## Materials and Methods

### Animals

Animals were group-housed at Baylor College of Medicine (BCM) on a 12 h light/dark cycle with ad libitum access to water and food. The mice used for viral studies were bearing a floxed VDR allele (VDR^f/f^) (18, 24). Additionally, we bred Sim1-cre mice (25) bearing a *Rosa26-tdTOMATO* allele onto the VDR^f/f^ mice. For the initial phenotyping experiments, mice bearing homozygous VDR floxed alleles were mated (VDR^f/f^ x VDR^f/f;Sim1-cre;TOMATO^), creating experimental PVH VDR knockdown mice (KD; VDR^f/f;Sim1-cre^) and control animals (VDR^f/f^), without regard for the presence or absence of the *TOMATO* gene. However, for electrophysiological experiments, we mated heterozygous VDR^f/+^ x VDR^f/+;Sim1-cre;TOMATO^ mice in order to have VDR^+/+;Sim1-cre;TOMATO^ controls which would allow for visualization of the PVH neurons. Animal numbers are stated in the figure legends. All studies used littermate controls. Mice were euthanized with ketamine/xylazine per BCM protocols. All studies were approved by the BCM Institutional Animal Care and Use Committee (IACUC).

### Diet

Animals were studied in a lean state (chow diet) or an obese state. Obesity was induced by a western diet (45% fat, D12451, Research Diets, New Brunswick, NJ) for 8 weeks prior to glucose tolerance testing.

### Virus Injection

Some mice at 8-12 weeks of age received 20nL of a replication-defective adenovirus-associated virus (AAV) containing Cre-recombinase (AAV9.CMV.HI.eGFP-Cre.WPRE.SV40 9.82e12 gc/mL; University of Pennsylvania Vector Core, Chapel Hill, NC) or its control (AAV-CMV-GFP-9 4.0e12 gc/mL) injected bilaterally into the PVN (coordinates 0.94A/P, 4.75D/V, 0.20M/L) as previously published (18).

### Glucose and Insulin Tolerance Tests

Intraperitoneal glucose tolerance tests (i.p. GTT) were performed as previously published (26). Mice were fasted for four hours and injected i.p. with 1.5 g/kg dextrose (D20W). A tail laceration was made, and blood glucose was measured on a glucometer in duplicate prior to dextrose administration (“0” minutes) and again 15, 30, 45, 60 and 120 min after dextrose administration. The insulin tolerance test (ITT) was performed similarly. Mice were fasted for four hours and injected i.p. with 1 unit/kg Humulin R U-100 (Eli Lilly, Indianapolis, IN). Blood glucose was measured from a tail vein laceration in duplicate on a glucometer prior to and at 15, 30, 45 and 60 minutes after insulin administration. Both GTT and ITT were performed in freely moving, conscious mice.

### Gene Expression

At euthanasia, the paraventricular hypothalamic nuclei were dissected through punch biopsy microscopically and rapidly frozen with liquid nitrogen. Tissue RNA was extracted using a Qiagen RNeasy kit. cDNA was isolated and real-time quantitative PCR (qPCR) was performed using a TaqMan 7900 sequence detection system with TaqMan universal PCR master mix and TaqMan gene expression assays (all from Applied Biosystems). Relative mRNA expression for the VDR using a primer/probe targeted to exons 3 and 4 of the VDR gene (Mm00437297_m1, Applied Biosystems) was calculated relative to the housekeeping gene *L32* using the ΔΔCT method. Quantification of mRNA expression was performed as previously described (27).

### Electrophysiology

Electrophysiology recordings were performed as previously published (26, 28, 29). Briefly, 10-12 week old Sim1-Cre;TdTOMATO mice were used where all neurons in the PVH would be labeled. Two male and female PVH VDR knockdown mice (VDR^f/f;Sim1-Cre;TdTomato^) and control mice (VDR^+/+;Sim1-cre;TOMATO^) were used for recordings. Separate mice were used for baseline and 1,25D_3_-activated recordings. Mice were anesthetized with isoflurane and brains were dissected rapidly and immersed in ice-cold and oxygenated cutting solutions (in mM: 10 NaCl, 195 Sucrose, 2.5 KCl, 1.25 NaH_2_PO_4_, 7 MgCl_2_, 25 NaHCO_3_, 5 glucose, 0.5 CaCl_2_, 2 sodium pyruvate. balanced with 95% O_2_/5% CO_2_). Coronal brain slices (220 μm) containing the PVH were cut with a Microm HM 650 V vibratome (Thermo Scientific) in oxygenated cutting solution. Slices were then incubated in oxygenated artificial cerebrospinal fluid (aCSF) (in mM: 126 NaCl, 2.5 KCl, 2.4 CaCl_2_, 1.2 NaH2PO_4_, 1.2 MgCl_2_, 11.1 glucose, and 21.4 NaHCO_3_, balanced with 95% O_2_/5% CO_2_, pH 7.4) to recover ~25 min at 32°C and subsequently for ≧1 h at room temperature before recording.

For whole-cell recording, slices were transferred to the recording chamber at room temperature and perfused continuously with aCSF bubbled with 95% O_2_/5% CO_2_ to ensure adequate oxygenation of slices. tdTomato^+^ neurons and tdTomato^-^ neurons were identified by using epifluorescence and IR-DIC imaging on an upright microscope (Eclipse FN-1, Nikon) equipped with a moveable stage (MP-285, Sutter Instrument). Patch pipettes with resistances of 3–5 MΩ were filled with intracellular solution (adjusted to pH 7.3) containing (in mM: 128 K gluconate, 10 KCl, 10 HEPES, 0.1 EGTA, 2 MgCl2, 0.3 Na-GTP and 3 Mg-ATP). Recordings were made using a MultiClamp 700B amplifier (Axon Instrument), sampled using Digidata 1440A and analyzed offline with pClamp 10.3 software (Axon Instrument). Series resistance was monitored during the recording, and the values were generally <10 MΩ and were not compensated. The liquid junction potential was +12.5 mV, and was corrected after the experiment. Data were excluded if the series resistance increased more than 20% during the experiment or without overshoot for action potential. Currents were amplified, filtered at 1 kHz, and digitized at 20 kHz. Current clamp was engaged to test neural firing frequency at the baseline and after puff delivery (Picospritzer III, Parker Hannifin) of VDR agonist 1,25-dihydroxyvitamin D_3_ (1,25D_3_) (500 ms at a concentration of 1 μM) (30). The resting membrane potential and firing frequency values were averaged within 2-min bin at the baseline or after 1,25D_3_ puff. In some experiments, the aCSF solution also contained 1 μm tetrodotoxin (TTX) and a cocktail of fast synaptic inhibitors, namely bicuculline (50 μM; a GABA receptor antagonist), D-AP5 (30 μM; an NMDA receptor antagonist) and CNQX (30 μM; an AMPA receptor antagonist) to block the majority of presynaptic inputs.

To measure mEPSC, the internal recording solution contained 125 mM CsCH3SO3, 10 mM CsCl, 5 mM NaCl, 2 mM MgCl2, 1 mM EGTA, 10 mM HEPES, 5 mM (Mg)ATP, 250 and 0.3 mM (Na)2GTP (pH 7.3 with NaOH) (31). The mEPSCs were recorded in whole-cell voltage-clamp mode, by holding the membrane potential a t Vh = −60 mV in the presence of 1μM TTX and 50 μM bicuculline.

### Statistics

For GTT, ITT, and body weight curves, data were analyzed with a paired 2-way ANOVA and Sidak *post-hoc* test. For AUC and qPCR measurements, data were analyzed with unpaired t-tests. The data for electrophysiology are presented individually for each recorded neuron and data were analyzed with unpaired 1-way ANOVA for baseline averages. Pre- and post-vitamin D treatment comparisons were made by paired 2-way ANOVA. P < 0.05 was considered to be statistically significant.

## Results

### Viral-Mediated PVH VDR Loss Does Not Impact Glucose Tolerance in Females

**Figure 1:**
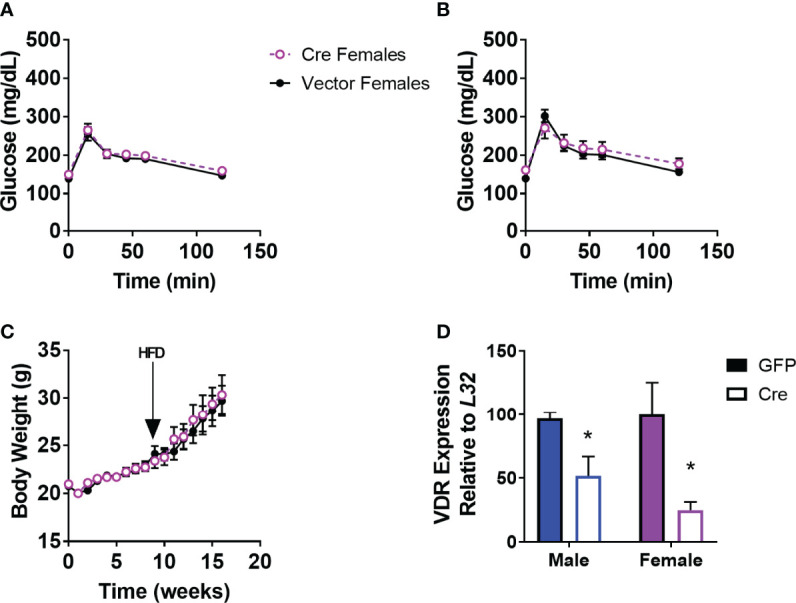
Viral Knockdown of VDR in the PVH of females does not alter body weight or glucose tolerance. **(A)** Glucose tolerance test in lean female mice (16-20 weeks of age) after AAV-control or AAV-Cre bilateral administration into the PVH (1.5g/kg). **(B)** Glucose tolerance test (1.5 g/kg) in 24-28 week old DIO mice. **(C)** Body weight trajectories of female mice after virus administration. HFD = start of high-fat diet to induce obesity. **(D)** VDR expression in the PVH in male mice (previously published) and females. n = 7-8/gp for **(C, D)** and 3-4/gp for **(D)** *p<0.05.

We demonstrated previously that male mice with PVH VDR viral knockdown had impaired glucose tolerance when obese, but not when lean (18). However, female mice with viral PVH VDR knockdown did not differ in glucose tolerance compared to control virus when lean (**Figure 1A**) or obese (**Figure 1B**). Importantly, these female mice were littermates to our previously published male cohort and thus, were of the same age and exposed to the same housing conditions as their male counterparts. Thus, external factors are unlikely to have influenced the observed sexual dimorphic difference in glucose tolerance. Furthermore, viral knockdown of VDR within the PVH in females did not alter their body weight in chow or DIO conditions (**Figure 1C**). We analyzed expression data from our previous male cohort and found that males and females had similar expression levels of VDR within the PVH and that virus administration resulted in a similar knockdown of the VDR (**Figure 1D**). Thus, the difference in glucose tolerance between male and female mice resulting from PVH VDR loss does not seem to be due to a difference in levels of the VDR but rather a sexually dimorphic function of the VDR within the PVH.

### Genetic-Mediated Loss of PVH VDR Disrupts Glucose Tolerance in Males but Not Females

**Figure 2:**
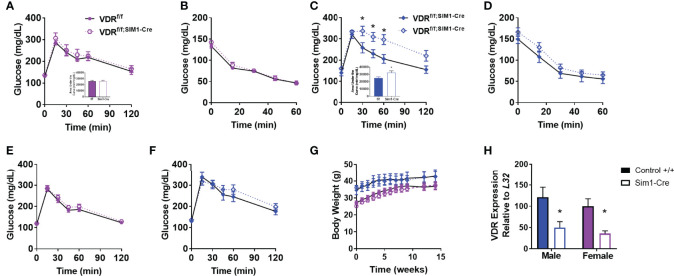
Genetic PVH VDR knockdown impairs glucose and insulin tolerance in males but not females. **(A, B)** Glucose tolerance **(A)** and area under the curve (inset) in 26-29 week old DIO female mice. **(B)** Insulin tolerance test in 30-33 week old DIO female mice. **(C)** Glucose tolerance test **(C)** and area under the curve (inset) in 30-33 week old DIO male mice. **(D)** Insulin tolerance test in 34-37 week old DIO male mice. **(E)** Glucose tolerance test in chow-fed female mice (18-21 weeks of age). **(F)** Glucose tolerance test in chow-fed male mice (22-25 week old). **(G)** Body weight trajectories of female and male mice after starting high-fat diet. **(H)** VDR mRNA expression in upper hypothalamus of male and female mice. N=8-14/gp for **(A–G)** and 4-6/gp for **(H)** Open shapes represent Cre+ mice; closed shapes represent control mice. *p<0.05 compared to same-sex control.

To determine if the effects of PVH VDRs were consistent across other models, we tested the effects of genetic deletion of PVH VDR. The single-minded homolog 1 gene (Sim1) is expressed significantly in the PVH and parts of the amygdala. By mating VDR^f/f^ mice with Sim1-Cre (25), we established mice with loss of VDR within the PVH from birth. Once again, PVH VDR knockdown did not result in any glucose or insulin tolerance abnormalities in females (**Figures 2A, B**). However, PVH VDR knockdown had a pronounced deleterious effect on glucose tolerance in males, as evidenced in their glucose curves (**Figure 2C**; main effect of genotype, p < 0.05) and AUC (**Figure 2C** inset). We did not observe significant insulin resistance during an ITT (**Figure 2D**). Before starting high-fat diet, mice were tested for glucose tolerance under chow-fed conditions. We observed no effect of PVH VDR loss in either sex when animals were on chow (**Figures 2E, F**), which is similar to our viral data shown in **Figure 1** and our prior publication (18). Body-weight trajectories on a high-fat diet did not differ between the groups (**Figure 2G**). VDR expression was significantly decreased in the PVH of VDR^f/f;Sim1-Cre^ mice but did not differ between sexes (**Figure 2H**).

### PVH VDR Loss Decreases Neuronal Activity in a Sex-Specific Manner

**Figure 3:**
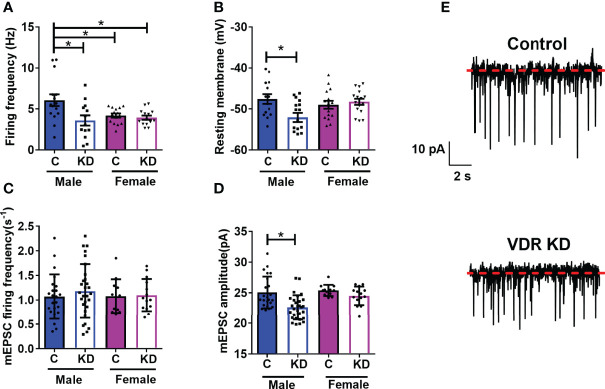
PVH VDR loss decreases neuronal activity in male but not female mice. **(A)** Firing frequency in male and female control VDR^+/+;Sim1-cre;TOMATO^ (“C”) and PVH VDR knockdown VDR^f/f;Sim1-cre;TOMATO^ (“KD”) mice. **(B)** Resting membrane potential. **(C)** mEPSC firing frequency. **(D)** mEPSC amplitude. **(E)** Representative traces from male control and PVH VDR knockdown mice. *p<0.05 *via* one-way ANOVA. N=12-30 neurons/group.

We and others have published that vitamin D has rapid actions on neuronal activity (18, 30, 32). However, whether VDRs affect basal levels of neuronal function within the hypothalamus were unknown. In DIO VDR^f/f^Sim1-Cre mice and their controls, we tested the role of VDRs on PVH neuronal function. Loss of PVH VDR decreased the basal firing frequency in male mice but not in female mice (**Figure 3A**). However, we noticed that female control mice had lower firing frequency compared to male controls. Loss of PVH VDR also hyperpolarized neuronal membranes in male but not female mice (**Figure 3B**). We did not observe differences between baseline levels in male and female controls, indicating a role for the VDR within the PVH on neuronal function that was apparent in males but not females. Since the loss of PVH VDR decreased neuronal firing frequency and hyperpolarized neurons, we tested whether loss of PVH VDR impacts miniature excitatory post- synaptic current (mEPSC), a key factor in the generation of action potentials. Interestingly, we did not find any effect of PVH VDR on the frequency of mEPSCs in either males or females (**Figures 3C, E**). Yet, male animals with PVH VDR loss had significantly lower mEPSC amplitude while females with PVH VDR loss had no effect (**Figures 3D, E**). These results indicate that VDR is required to maintain normal post-synaptic responses of male PVH neurons to excitatory inputs (likely *via* glutamate), but female PVH neurons do not require this VDR function.

### PVH Neurons Respond to 1,25D3 Through the Vitamin D Receptor in Both Males and Females

**Figure 4:**
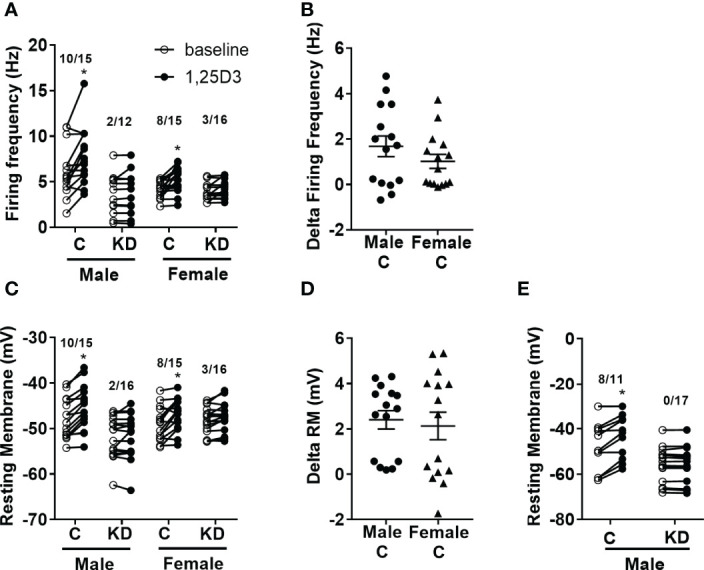
Vitamin D activates PVH neurons through the VDR. **(A)** Firing frequency response to 1,25D_3_ treatment in male and female control VDR^+/+;Sim1-cre;TOMATO^ (“C”) and PVH VDR knockdown VDR^f/f;Sim1-cre;TOMATO^ (“KD”) mice. **(B)** Firing frequency change after 1,25D_3_ in male and female control mice. **(C)** Resting membrane potential response to 1,25D_3_ treatment. **(D)** Resting membrane change after 1,25D_3_ in male and female control mice. **(E)** Resting membrane potential response to 1,25D_3_ treatment in male mice in the presence of synaptic blockers (TTX, DAP-IV, CNQX, and bicuculine). *p < 0.01 compared to untreated state. N=11-18 neurons/group.

We previously published that vitamin D has acute activating effects on neurons in the hypothalamus (18, 26). Thus, we next tested the loss of the VDR within the PVH had any sex-specific effects on vitamin D-mediated actions on electrical activity. Active vitamin D (1,25-dihydroxyvitamin D3; 1,25D_3_) increased the firing frequency in 10 of 15 (66.7%) PVH neurons (identified by Sim1-Cre mediated Tomato florescence) in males and 8 of 15 (53.3%) PVH neurons in females (**Figure 4A**). However, vitamin D had reduced effects in mice with PVH VDR KD, only activating 2 of 12 (16.5%) male neurons and 3 of 16 (18.7%) female neurons. There was no difference in the magnitude of change in firing frequency after 1,25D_3_ stimulation in control male and female mice (**Figure 4B**). Similarly, 1,25D3 depolarized 10 of 15 PVH neurons in males and 8 of 15 PVH neurons in females (**Figure 4C**). Loss of PVH VDR neurons decreased the ability of 1,25D3 to depolarize PVH neurons. Only 2 of 16 neurons in males and 3 of 16 neurons in females were depolarized by vitamin D in animals with PVH VDR knockdown (**Figure 4C**). There was no difference in the change in resting membrane potential between the sexes (**Figure 4D**). To confirm that the effects of vitamin D on neuronal function were due to direct actions of vitamin D on these PVH neurons, we treated neurons from male mice with vitamin D (1,25D_3_) in the presence of synaptic blockers. We found that in the presence of synaptic blockade, vitamin D depolarized 8 of 11 (72.7%) PVH neurons in control animals and was unable to depolarize any neurons in animals with PVH knockdown (**Figure 4E**). This data indicated that vitamin D (1,25D_3_) directly acts on VDR in PVH SIM-1 neurons to activate their activities.

## Discussion

Our results show a consistent requirement for PVH VDR in male mice for glucose regulation, but not in female mice. Human epidemiological data has recently supported the possibility of a sex-specific effect of vitamin D deficiency on glucose regulation (19–22, 33). However, there are undoubtedly other analyses where no mention of sex-specific effects exists (34–37). One possibility for these differences may be that the impact of sex on glucose homeostasis negates the ability to see sex-specific interactions with vitamin D and glucose in some studies. Given that we only see the effects of PVH VDR in DIO mice, and the mediators of this are unknown, study populations may differ in their underlying characteristics that are necessary to observe the effects of vitamin D action in the brain on glucose homeostasis.

Our previous (18) and current data show no effect of PVH VDR on body weight during the development of obesity. This is intriguing given the very powerful impact effect of the PVH on body weight. We recently created and published a novel VDR-Cre mouse to visualize VDR positive neurons in the PVH (26). We did not observe significant colocalization of VDR PVH neurons with oxytocin or vasopressin in that study. Given this study, we surmise that there is likely little colocalization with MC4R neurons. While more work needs to be done to characterize PVH VDR neurons, their ability to regulate glucose independent of weight indicates that they likely represent an important, and unique, neuronal population.

We again found a requirement for a DIO state for animals to display the effects of PVH VDR loss on glucose homeostasis. A DIO animal is known to have hypothalamic inflammation (38). Given that vitamin D has known anti-inflammatory properties, it may be that the function of VDR within the hypothalamus is to aid in decreasing inflammation. However, there is also a possible interaction between components of our DIO-inducing diet and VDR, which causes effects of PVH VDR loss only in a DIO state. Determining the mechanisms behind this interaction between weight status/diet and VDR action has clear clinical relevance given the strong association of obesity and a western diet with insulin resistance and diabetes.

We have presumed that the effects observed in our VDR^f/f^-Sim1-Cre mice are due to the effects of PVH VDR. However, this mouse model would also have effects of VDR knockdown within the amygdala (25). Some data show that insulin signaling in the amygdala may affect peripheral glucose tolerance (39). Thus, it is possible that some of the effects of our Sim1-Cre mediated VDR knockdown are through the amygdala. However, since both direct PVH VDR knockdown with a virus and genetic VDR knockdown resulted in similar abnormalities in glucose tolerance, effects of the amygdala are likely minor in this group. Future studies will need to confirm if VDR action in the amygdala has any impact on glucose tolerance.

In these experiments, some control PVH neurons did not respond to vitamin D. However, we were not able to identify VDR positive neurons. Fluorescent neurons indicated the presence of Sim1 expression, a marker for PVH neurons. Thus, the lack of response to vitamin D in some of the PVH neurons is likely due to a lack of VDR within those neurons. This is supported by our recent work demonstrating that all VDR-Cre reporter neurons in our new mouse model responded to 1,25D_3_ while no non-reporter neuron responded (26). Additionally, a few neurons in the PVH VDR knockdown group responded to vitamin D. Given that we did not see a complete loss of all VDR expression, consistent with other cre models, this is likely from some VDR expression present in those neurons.

The loss of effect of vitamin D in our PVH VDR knockdown group supports the specificity of the VDR in mediating the effects of vitamin D overall. Our electrophysiological experiments use higher than physiological concentrations since the drug is quickly washed away. However, given that the electrophysiological recording of neurons lacking the PVH VDR, and thus lacking endogenous vitamin D at physiological levels, is opposite of our pharmacologic study adding vitamin D, it is likely that the experiment represents actual responses of *in vivo* vitamin D.

This paper demonstrates that the VDR itself affects neuronal activity as loss of PVH VDR significantly impairs the firing frequency, resting membrane potential, and mEPSC amplitude in male mice. We did not observe any effect of PVH VDR loss on any parameter in females, although the impact on firing frequency may be masked by the overall lower firing frequency observed in our female controls. However, given the lack of effect of PVH VDR loss in females on the other parameters, where differences from male controls were not observed, our data strongly support a sex-specific effect of PVH VDR loss on neuronal activity that may explain our underlying sex-specific glucose phenotype.

Given that we observe a detrimental effect of VDR loss on postsynaptic excitatory response, these effects may be secondary to genomic actions of the VDR on the glutamatergic system. There is limited data on the role of vitamin D on the glutamatergic system. A previous group published that vitamin D supplementation altered the expression of glutamate receptor subunits in diabetic rats (AMPA GluR2 and GluR4) (40). Additionally, we previously published that hypothalamic tissue from DIO male rats treated with 1,25D3 had increased expression of two glutamate receptor subunits *Grin2a* and *Grin2b *(30). A few studies show vitamin D is protective against glutamate-induced neurotoxicity (41, 42), which may be due to vitamin D’s known anti-inflammatory properties. These articles did not measure the effects of vitamin D on glutamate receptors. One study performed transcriptomic analysis of a mixed neuron-glial cell culture of neural stem cells but only found 27 genes differentially regulated by vitamin D, and none were glutamate receptor subunits (43). However, this may be due to the differences between embryonic and adult gene transcription. Altogether, our data and previously published data would support that vitamin D likely has genomic effects in neurons to support baseline electrical activity and the ability to respond to circulating 1,25D_3_ rapidly.

An interesting finding in our study is that despite the sex-specific differences in the glucose phenotype and electrical activity of PVH neurons in mice with PVH VDR loss, we did not observe sex-specific differences in the ability of vitamin d to activate PVH VDR neurons. This is consistent with our previous study demonstrating similar effects of 1,25D_3_ to activate VDR-Cre reporter neurons (26). This may mean that vitamin D can be a helpful treatment in both sexes for neurological disorders. Since we only investigated the sex-specific effects of the VDR within the PVH, more work is needed to determine differences within other brain areas, especially regarding vitamin D treatment. Additionally, we did not test how global vitamin D deficiency may impact neuronal function of VDR^+^ neurons or the effects of 1,25D_3_ on neuronal activity. Thus, there is future work needed to understand how the VDR function in the brain is correlated with physiologic measures of vitamin D action (i.e. low 25-hydroxyvitamin D).

Overall, this study strongly demonstrates that PVH VDR has sex and weight status-specific actions. In two different mouse models, loss of PVH VDR results in glucose intolerance in DIO males, but not in DIO females, nor in lean mice from either sex. Additionally, loss of PVH VDR diminishes baseline neuronal firing, resting membrane potential, and mEPSC amplitude in male but not female mice. However, neurons from both sexes responded well to exogenous vitamin D, in a VDR-dependent manner. Thus, this may explain some of the clinical data demonstrating associations between vitamin D deficiency and glucose levels in men but not women. The clear effect of vitamin D in the brain to regulate glucose levels indicates a need to understand the extent to which this action is necessary for exogenous vitamin D to impact glucose regulation and how this may be modified by sex or dietary factors.

## Data Availability Statement

The raw data supporting the conclusions of this article will be made available by the authors, without undue reservation.

## Ethics Statement

The animal study was reviewed and approved by Baylor College of Medicine Institutional Animal Care and Use Committee.

## Author Contributions 

All authors listed have made a substantial, direct, and intellectual contribution to the work, and approved it for publication.

## Funding

This work was supported by the US Department of Agriculture, Agriculture Research Service (cooperative agreement No. 58-6250-6-001 to SS), Baylor College of Medicine Pediatric Pilot grant to SS, and the American Diabetes Association (1-17-JDF-037 to SS).

## Conflict of Interest

SS receives support from Rhythm Pharmaceuticals for scientific advisory boards and for Speakers Bureau engagements.

The remaining authors declare that the research was conducted in the absence of any commercial or financial relationships that could be construed as a potential conflict of interest.

## Publisher’s Note

All claims expressed in this article are solely those of the authors and do not necessarily represent those of their affiliated organizations, or those of the publisher, the editors and the reviewers. Any product that may be evaluated in this article, or claim that may be made by its manufacturer, is not guaranteed or endorsed by the publisher.
